# Working conditions in primary care: a qualitative interview study with physicians in Sweden informed by the Effort-Reward-Imbalance model

**DOI:** 10.1186/s12875-021-01500-1

**Published:** 2021-07-10

**Authors:** Per Nilsen, Hanna Fernemark, Ida Seing, Kristina Schildmeijer, Carin Ericsson, Janna Skagerström

**Affiliations:** 1 Department of Health, Medicine and Caring Sciences, Division of Health and Society, Linköping University, 581 83 Linköping, Sweden; 2 Department of Behavioral Science and Learning, Linköping University, 581 83 Linköping, Sweden; 3 Department of Health and Caring Sciences, Faculty of Health and Life Sciences, Linnaeus University, 391 82 Kalmar, Sweden; 4 Medicine Center, Region Östergötland, 581 85 Linköping, Sweden; 5 Research and Development Unit in Region Östergötland, Linköping, Sweden

**Keywords:** Primary care, Physicians, Working conditions, Job satisfaction, Efforts, Rewards, Over-commitment

## Abstract

**Background:**

Many problems with primary care physicians’ psychosocial working conditions have been documented. Many studies on working condition have used the Effort-Reward-Imbalance (ERI) model, which posits that poor health and well-being may result from imbalances between the level of effort employees perceive that they put into their work and the rewards they receive. The model has not been used in qualitative research or applied to investigate primary care physicians’ working conditions. The aim of this study was to apply the ERI model to explore the perceived efforts and rewards by primary care physicians in Sweden and approaches they take to cope with potential imbalances between these efforts and rewards.

**Methods:**

The study has a qualitative design, using semi-structured interviews. A purposeful sampling strategy was used to achieve a heterogeneous sample of primary care physicians who represented a broad spectrum of experiences and perceptions. We recruited 21 physicians; 15 were employed in public health care and 6 by private health care companies.

**Results:**

The analysis of the interviews yielded 11 sub-categories: 6 were mapped to the efforts category, 3 were attributed to the rewards category and 2 were approaches to coping with effort/reward imbalances. Many of the statements concerned efforts in the form of high workload, restricted autonomy and administrative work burden. They also perceived resource restrictions, unpredictability of work and high expectations in their role as physicians as efforts. Three types of rewards emerged; the physicians found their job to be stimulating and meaningful, and the work climate to be supportive. The physicians coped with imbalances by means of job enrichment and using decisional latitude.

**Conclusions:**

Primary care physicians perceive numerous types of efforts in their job, which is consistent with research concerning work stress and associated consequences, such as poor subjective health and well-being. There are also rewards according to primary care physicians, but the findings suggest a lack of reciprocity in terms of efforts and rewards although firm conclusions cannot be drawn since the study did not investigate the magnitude of the various efforts and rewards or the effectiveness of the approaches the physicians use to cope with imbalances. The ERI model was found to be useful to explore physicians’ primary care work and working conditions but its applicability likely depends on the type of work or professions being studied.

## Introduction

Health care is often a stressful work environment. Physicians are an important risk group for whom work stress has become an increasing concern.

Primary care research in several countries has documented many work-related health problems among physicians. Studies have identified problems with various working conditions, including low perceived compensation and social status compared with other specialist fields in medicine, lack of recognition for good work, large workload, low work commitment, poor job satisfaction, high staff turnover and difficulties with recruiting primary care physicians [1–18]. As first-line care, primary care is vulnerable to changing societal conditions that likely affect the working conditions, including ageing populations, higher patient expectations for access to care and increased patient involvement in care decision making [19].

Problems with the psychosocial working conditions in primary care in Sweden have received increased attention in the past decade. There has been considerable debate among the physician profession, policymakers and researchers concerning the prevalence and causes of stressful work environments in primary care [20, 21]. Swedish primary care physicians have reported experiencing negative psychosocial working conditions and work-related stress more frequently than other professions in primary care [6, 22]. There is a shortage of physicians in Swedish primary care, including specialist and resident physicians, and difficulties in recruiting and retaining physicians have been documented [23]. Compared with most other European countries, Sweden has a low proportion of primary care physicians versus hospital physicians [24]. From an international perspective, primary care physicians in Sweden have been shown to be more dissatisfied with the health care system than their colleagues in many western countries [25].

The Swedish health care system consists of 21 regions providing health care for the Swedish population of more than 10 million funded primarily by taxes. All residents are insured by the state with equal access to health care for the whole population. Fees are low and regulated by law [26, 27]. Primary care is first-line care in Sweden and is responsible for the delivery of basic medical treatment, preventive work and rehabilitation. There are approximately 1200 primary care units in Sweden of which 43% are privately owned. The private health care companies are usually contracted to regions and the out-of-pocket fees for their patients are equal to that of publicly funded health care [26]. Primary care units typically employ physicians, nurses, physiotherapists and psychologists, although there are variations in the composition of the workforce among different units [28].

Psychosocial working conditions in many settings have been studied using the Effort-Reward-Imbalance (ERI) model [29]. The model is used to identify a potential mismatch between efforts and rewards in a work setting and has had considerable success in predicting the health status of employees [30, 31]. It postulates that poor health and well-being may result from imbalances between the level of effort employees perceive that they put into their work (e.g. due to having a considerable workload and overtime work) and the rewards they receive (e.g. in terms of having good promotion prospects and secure employment) [29]. The underlying theoretical principle of ERI is the notion of social reciprocity, which posits that individuals invest efforts and expect rewards in return. Failed reciprocity resulting from a violation of this norm of return expectancy elicits negative emotions and stress responses [31].

The ERI model has been applied in many cross-sectional survey studies of working conditions in various countries, health care settings and professions. However, there is a paucity of research using the model to study working conditions in primary care or specifically amongst physicians in this setting. We have only found one study by Teles et al. [9] that applied the ERI model to investigate working conditions among primary care physicians, but they integrated physicians into their sample of 729 Brazilian health care workers (including nurses, dentists, community health workers, etc.) without providing separate results for the different professions. The ERI model has been used in studies of nurses [4] and physician assistants [1] and in studies in hospitals [11] and secondary public health care facilities [8].

Further, the ERI model has only been used in quantitative survey research, although the model could also guide qualitative research to gain a deeper understanding of how physicians in primary care make sense of efforts, rewards and what approaches they use to cope with potential imbalances between efforts and rewards. Addressing the topic inductively, by posing open questions that allow for physicians’ exploration, rather than asking them to choose between fixed response options in a questionnaire, could yield new insights into their views on efforts and rewards in their primary care work. A qualitative approach could also facilitate new insights into how perceived effort/reward inequity might be overcome.

Addressing these knowledge gaps, the aim of this study was to apply the ERI model to explore the perceived job-related efforts and rewards by primary care physicians in Sweden and approaches they take to cope with potential imbalances between these efforts and rewards. It is important to investigate their attitudes towards primary care work, both the work itself and working conditions, to gain an in-depth understanding of what types of changes might be needed to improve working conditions in primary care to reduce work-related health problems and to make primary care work a more appealing career option.

## Methods

### Study design

The study has a qualitative design, using semi-structured interviews. A qualitative approach with interviews was considered relevant to gain a deep understanding of primary care work and working conditions based on physicians’ experiences and perceptions. All participants in the interviews were employed in primary care. The research team behind the study was comprised of a behavioural economist (PN), a physician (HF), a political scientist (IS), a registered nurse (KS), a behavioural scientist (CE) and a public health researcher (JS).

### Recruitment of participants

A purposeful sampling strategy was used to achieve a heterogeneous sample of participants [32]. We recruited physicians who: (1) were employed in publicly funded primary care units and in private health companies; (2) were employed in primary care units that differed with regard to geographic location; (3) were specialists and residents in primary care; and (4) currently worked with conventional face-to-face patient consultations although they may also be active in digital consultations, which have increased rapidly in Sweden in recent years [19]. The objective of this sampling strategy was to recruit physicians who represented a broad spectrum of experiences and perceptions of relevance for exploring the working conditions in primary care.

We recruited 21 primary care physicians for the interviews. Of these, 15 were employed in public health care. To recruit these participants we contacted all 21 regions in Sweden by examining the regions’ websites to identify the person who seemed to be responsible for digital consultation in the region because we wanted to involve physicians who had experience with both digital and conventional face-to-face consultations. We sent an e-mail to this person, briefly informing them about our study and asking for physicians from the region to participate. We did not receive any response from 8 regions; 4 regions agreed to participate and provided contact information for physicians who had worked with digital consultation. We approached 29 primary care physicians from the 4 regions, and 15 who fulfilled the four purposive sampling criteria (see above) agreed to participate.

We recruited 6 participants working in private health care. To this end, we approached 7 private companies. Of these, 5 agreed to participate in our study. We approached 12 physicians from these companies, and 6 who fulfilled the four purposive sampling criteria agreed to participate. All the participants had some experience of employment in publicly funded health care.

The research was conducted in accordance with the Declaration of Helsinki. The study was approved by the Regional Ethics Review Board in Linköping (2019–01,910). Transcripts are stored in the authors’ password-protected computers and no unauthorized persons have access to the data.

### Data collection

The authors developed a semi-structured interview guide to capture the physicians’ perceptions and experiences concerning their psychosocial work environment. The interview guide was assembled by the research team behind the study, based on the existing literature on psychosocial work environments. The questions were informed by the ERI model, but were not constructed to be an oral version of survey questionnaires based on ERI. Rather, the ambition was to formulate broad, open-ended questions that could capture influences of working conditions on the participants’ job satisfaction. The questions concerned the physicians’ conventional face-to-face patient consultations and their work with digital consultations [19].

The questions that were analysed in this study were the following: Why did you choose the physician profession and to work as a primary care physician? What is most important for you in your role as a physician? How do you perceive the work situation to be at your primary care unit, in terms of working conditions, workload and expectations on you? How flexible do you perceive your job to be? Would you like it to be different in any way and, if so, how? What support do you receive from the management or manager? How is the collegial support and collaboration at your primary care unit? Is there anything you would like to see more or less of? How do you maintain the balance between your private and working lives? Numerous probes and follow-up questions were also asked, e.g. what the physicians considered to be the best feature of their work, how satisfied they were with the current work environment and how their working conditions had changed over time.

We pilot tested the interview guide in 2 interviews, which indicated that further questions regarding aspects of digital work needed to be incorporated into the interview guide. Despite this, the first 2 interviews included relevant information and were therefore included in the analysis.

The interviews were conducted by all authors except PN and JS. Each interview lasted between 24 and 84 min and was digitally audio recorded. No field notes were taken during or after the interviews. The interviews were conducted by video meeting, telephone or a personal meeting, depending on what suited the participant best. Before the interviews were conducted, the participants signed informed consent stating that their confidentiality was guaranteed and that no one other than the interviewer would know their identity. To the other researchers, the participant was known only by initials and other demographic, non-identifying data. No participant withdrew participation during or after the interviews.

Only the participant and interviewer were present during the interviews to allow the participant to speak freely. The participants did not have any previous relationship with the researchers except for the 3 participants who were known to HF: 2 participants in the pilot interviews and one participant in the subsequent interviews. The first 3 interviews were transcribed verbatim by HF and the remaining interviews were transcribed by a professional transcription agency. All transcripts were carefully examined by HF to ensure accuracy. The interviews took place from April to October 2019.

### Theoretical framework

We used the ERI model as an analytical tool, i.e. as a framework for a qualitative directed content analysis [33] of the interviews with regard to efforts and rewards experienced and/or perceived by the physicians and their approaches to coping with imbalances that may exist between efforts and rewards. Efforts refer to job-related factors that are imposed on the employee and make work demanding, e.g. time pressure due to a heavy workload, interruptions while performing the job, a great deal of job responsibility and pressure to work overtime. Rewards can be job-related factors such as receiving adequate salary, good promotion prospects, secure employment, a position that adequately reflects a person’s education and training, respect from superiors and/or other relevant persons, adequate support in difficult situations and being treated fairly at work. The ERI model posits that individuals use different approaches to cope with effort/reward imbalances, referred to as over-commitment, to modify deleterious effects on health and well-being, e.g. sacrificing a great deal for one’s work and seeking approval [34].

In this study, efforts were work-related characteristics (e.g. terms, responsibilities and circumstances) that were perceived to have a negative impact on the physicians’ job satisfaction, rewards were characteristics that were perceived to positively influence job satisfaction and approaches to coping with effort/reward imbalances were personal strategies used by the physicians to improve job satisfaction. Job satisfaction is the positive and negative attitudes employees have towards their work or individual aspects of the work, encompassing both the work itself and the working conditions [35].

### Data analysis

Participants’ responses concerning job-related efforts and rewards and their approaches to coping with effort/reward imbalances were analysed using directed content analysis, applying the 3 predetermined categories of the ERI model (i.e. efforts, rewards, approaches to cope with effort/reward imbalances) to develop the initial coding scheme. Directed (or deductive) content analysis involves the use of existing categories from a theory or framework to guide the data analysis. In contrast, in inductive (or conventional) content analysis the categories emerge from the process of data analysis [33].

All authors read all transcripts to obtain an understanding of the whole and examined the ERI model since the three categories of the model provided the framework for the directed content analysis. In the first step, PN coded the transcripts by identifying participants’ statements that were related to one of the three ERI categories. The statements were grouped into meaning units (i.e. constellations of statements that relate to the same central meaning), which were assembled into sub-categories that shared content associated with any of the three ERI categories.

The sub-categories were created to be internally homogeneous and externally heterogeneous and were intended to be mutually exclusive [36]. Each sub-category was given a name to provide a concise description of what it refers to and a description was generated to provide information about what is meant by a given sub-category [36].

In the next step of the analysis, PN mapped each sub-category onto one of the three pre-determined categories (efforts, rewards or coping approaches). This step involved all authors reading and reflecting on the three ERI-related categories and the proposed sub-categories, including their names, descriptions and associated quotations. These findings were discussed at several Zoom meetings (the analysis was carried out during onsite workplace restrictions due to the coronavirus pandemic) and via emails. This process continued until consensus was reached on the categories and sub-categories.

Representative quotations from participants were selected by PN and HF and were then discussed with the rest of the team before the final quotations were agreed upon. Quotations are marked from physician #1 to physician #21 in the Results.

## Results

The characteristics of the 21 participants are shown in Table 1. Seventeen of the participants were employed by primary care units in 4 regions and 11 worked in 5 different private companies. Participating regions and companies were in central and southern Sweden. Ten of the participants had received their medical training in Sweden and 11 had undergone medical education abroad.

**Table 1:** _Participant characteristics_

Characteristic	Number (%)
**Sex**	
Male	10 (48)
Female	11 (52)
**Age**	
30–39 years	7 (33)
40–49 years	7 (33)
50–63 years	7 (33)
**Level of medical training**	
Specialist in primary care medicine	16 (76)
Resident in primary care medicine^a^	5 (24)
**Employer**	
Region	15 (71)
Private company	6 (29)

^**a**^In Sweden, resident physicians have finished medical school, possess a medical licence and for 5 years, they provide health care and are learning to become specialist physicians (in this case, specialists in primary care medicine)

**Fig. 1:**
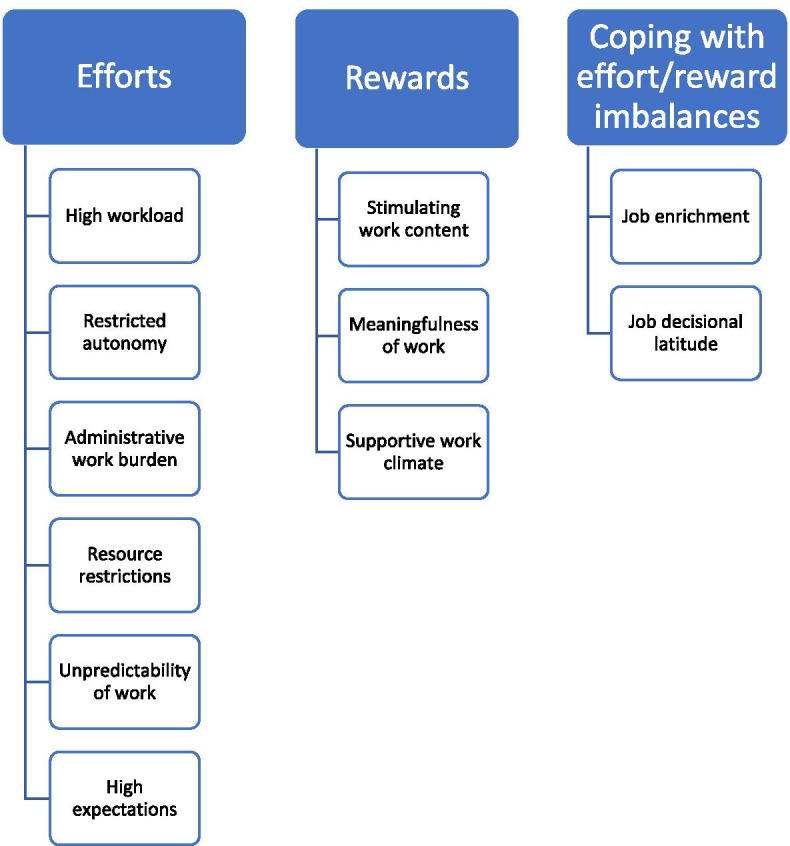
Categories based on the ERI model and associated sub-categories

The analysis of the interviews yielded a total of 11 sub-categories: 6 were mapped to the efforts category, 3 were attributed to the rewards category and 2 concerned approaches to coping with effort/reward imbalances (Fig. 1).

### Efforts

#### High workload

Physicians described their workload as high, which had a negative impact on their job satisfaction. Many also said that the work burden was uneven, with busy, intense days interchanged with calmer periods. Stressful workdays could affect them long after work was over for the day. It was even argued that the high workload was too exhaustive to work full-time as a clinician in primary care. Reasons for the high and/or uneven workload, according to the physicians, included high staff turnover that resulted in understaffing in relation to the number of patients listed at the unit and a general trend towards increased responsibilities for primary care to perform tasks previously handled in secondary care.

*I had such a day today. I was at the health centre all day until 4 [pm], I came home, I thought, 'No, now my head is cracking.’ There were so many questions, tasks in the journal, extra prescriptions, extra notes on the table. [#17]*

*It is so intense that you cannot bear it. But we have many who do something else besides, work at the university with teaching or research or so. [#20]*

There were also physicians who described their workload in a more favourable light, emphasizing that the amount of work was usually reasonable although it could still vary a great deal from day to day or with regard to longer time periods.

*[The workload is] good, although there are periods during the year when it is tougher. [#1]*

*It varies. Some days, very reasonable, other days, long queues, many patients who need help. [#11]*

#### Restricted autonomy

The physicians’ job satisfaction was negatively influenced by what they perceived as restricted autonomy. They described having limited decisional latitude and influence over their work. Many of the statements specifically concerned dissatisfaction with the regimented nature of the work and the lack of flexibility it allowed. The lack of independence was attributed to the governance of health care; many physicians were negative about higher management and political levels dictating terms for primary care. Physicians expressed that they felt controlled and believed many tasks that were imposed on them detracted from their desired focus on caring for patients.

*We work so damned unstructured because we cannot control ourselves. All these ideas from above make it harder to manage. The big change was 15 years ago, when ‘silo governance’ was introduced. Previously I was more self-governing, then they introduced the silos and they must have statistics. [#4]*

*It's quite inflexible. The schedule is set, it is often fully booked and it is difficult, especially when you have other assignments and would need to take time off. [#8]*

#### Administrative work burden

The administrative work tasks imposed on the physicians led to many complaints and had a negative impact on their job satisfaction. Their main concern was that this type of work was time-consuming for which they did not seem motivated, and it ultimately detracted from a desired patient focus. The physicians mostly spoke about the consequences of the administrative work burden, but one of them blamed New Public Management principles for this development.

*I would like to spend a little less time on administration. It's a lot to sit and write letters or write medical certificates and stuff like that. That's the part you really want to shorten. [#7]*

*You meet patients, but then every patient also requires administration, so it is usually difficult to keep up with it. There is often too little time for the administrative tasks because we don’t have enough doctors. [#16]*

#### Resource restrictions

Job satisfaction was also negatively affected by perceived resource limitations. The physicians mentioned restrictions concerning technology, transfer of data and information as well as with the localities. They believed that these problems could have a negative impact on the quality and effectiveness of the work they perform.

*We are very vulnerable to IT problems, which we had here this morning. There were several employees who could not log in at all to the system. It is difficult to catch up later. These are probably our biggest challenges, IT problems and congestion in the [primary care unit] premises. [#19]*

*We do not have such a good structure for information transfer; a lot of mails that come in duplicate. Yes, it’s difficult. [#21]*

#### Unpredictability of work

The physicians’ job satisfaction was negatively influenced by difficulties they perceived with regard to planning work ahead and being prepared for unexpected events that might occur. The physicians accepted the inherent “putting out fires” nature of much health care, yet they were dissatisfied with the focus on the short-term as it impeded their ability to plan and perform their work as well as they would like.

*When we work in health care, care in general, planning is very difficult. You notice this quite clearly because there is a lot that happens unforeseen in terms of patient flow. And we are quite vulnerable when there are fewer staff due to diseases and so on. [#3]*

*There are both calm days and stressful days, but I would say that days are mostly stressful. If bookings are wrong, it affects the day a lot. Then it will be stressful of course. [#9]*

#### High expectations

Some of the expectations the physicians associated with their role as physicians had a negative impact on their job satisfaction. Although they recognized that they should be held accountable for decisions they make, they believed it was often difficult to live up to moral, ethical and patient safety ideals under less than optimal working conditions. Managers, colleagues and patients all contributed to the high expectations the physicians felt in their work.

*There is an expectation from my employer that I will do a good job and that the patients, above all, will be satisfied. There is a lot of focus on this and I think it is very good. And of course there is an expectation that I will ‘produce’. [#11]*

*There are more expectations from the patients. The patients are more well-read and I think that is good, I like that the patients have read online and that makes them better prepared, but they also can make justified demands. It can be perceived as pressing for some, that they know what they need. [#21]*

### Rewards

#### Stimulating work content

The stimulating content of the work the physicians do positively influenced their job satisfaction. They particularly appreciated the interesting challenges provided with the variation and breadth of tasks in primary care work, which required them to develop and use many different skills and abilities. Meeting, getting to know and following patients over time were mentioned as inspirational and satisfactory aspects of their work.

*The variety [is the best part of the job], I would say. I am a person who gets a little tired if I have to do the same thing all the time. [#9]*

*I chose primary care because I’d like to follow my patients over time and develop a sort of relationship with the patients as they return to their primary care unit. [#11]*

#### Meaningfulness of work

Physicians considered their work to be highly meaningful, which contributed positively to their job satisfaction. They recognized that the work they perform as physicians is of great importance for patients who seek primary care for help with their illnesses. Having the skills and ability to make a difference by helping patients and achieving patient benefits was important for the physicians’ sense of meaningfulness. With few exceptions, the physicians did not mention financial aspects as being relevant for the meaningfulness of work.

*The patient contact is probably the most important because that is why I became a clinician. The patient contact gives a lot back. [#14]*

*[Most important in my work] is the patient contact, to have a valuable and good time together with the patient that is valuable for the patient. But it also rewarding for me as a doctor that I can help the person who is seeking help from me. [#18]*

#### Supportive work climate

Working in a supportive climate at a primary care unit had a positive impact on the physicians’ job satisfaction. Collaborating with other physicians and staff from other professions, receiving support from colleagues and interacting with and receiving feedback from patients were important aspects of the favourable work climate. The opportunity to speak informally with and ask other physicians was also appreciated. Some physicians claimed that job satisfaction was primarily due to the social relationships at work.

*The best thing about my job, hand on heart, may not be the medical work itself, but it is probably this togetherness we have with my employees and colleagues and with other professions. [#3]*

*It has become a good learning climate. There are many who are willing to share their knowledge so we can increase our overall competence level. [#19]*

### Approaches to cope with effort/reward imbalances

#### Job enrichment

The physicians utilized various opportunities to enrich their job as a way to cope with imbalances between efforts and rewards, thus improving their job satisfaction. This involved initiatives to diversify their work tasks, develop new competences and take on responsibilities beyond the normal job in primary care. Such initiatives could reduce the workload because it gave them a break from regular clinical patient work in the primary care unit.

*I just felt, ‘I cannot live like this.’ It became far too much [work] and we are understaffed and there was never enough [time and staff]. When this online work appeared, I thought I would give it a try. This has saved me, so I have been able to continue working as much as before. I have my quality of life and work quality too. [#17]*

*There are of course always exciting development issues. I have not been involved in the introduction of this digital application, the AI function. It would have been fun to have been more involved in it, but you cannot be everywhere. I would like to get more involved in medical quality issues. [#21]*

#### Job decisional latitude

Using job decisional latitude to influence one’s own work schedule, i.e. when and how much to work, provided the physicians with another means to reduce an overbearing workload and try to avoid over-commitment in their work, thus improving their job satisfaction. Several physicians described the attainment of a good work/leisure balance as a difficult struggle.

*You have to fight to catch up and I'm used to it now. I can set limits, but [to maintain a decent work/leisure balance] that's tough. [#4]*

*You have to work actively to get it [balance between work and leisure]. The job can devour all your time, that happens fast, because you get a lot of assignments all the time. So somewhere you proactively just have to prioritize well and above all try to make time for your own life so that you do not get stressed by your job. [#6]*

## Discussion

This study sought to explore primary care physicians’ perceived job-related efforts and rewards as well as their approaches to coping with potential imbalances between efforts and rewards. We used the three categories of the ERI model (i.e. efforts, rewards and approaches to coping with effort/reward imbalances) as a framework for the analysis of interviews with the physicians. Most of the sub-categories that emerged from the analysis could be mapped to the efforts category of the ERI model: high workload, restricted autonomy, administrative work burden, resource restrictions, unpredictability of work and high expectations in their role as physicians. These findings are consistent with previous primary care research concerning work stress and associated consequences, such as poor subjective health and well-being [3–11]. The physicians also perceived rewards in their job, but the findings suggest a lack of reciprocity in terms of efforts and rewards. However, firm conclusions about an effort/reward imbalance cannot be drawn since the study did not investigate the magnitude of the various efforts and rewards or the effectiveness of the approaches the physicians used to cope with imbalances. Most of the physicians lamented about their high workload although there were also those who described the work burden in more neutral terms, emphasizing that their work was also characterized by calmer periods. The overall findings concerning the work burden are consistent with other studies that have documented problems with high workload in primary care in many countries [24, 37, 38]. High workload in primary care has been attributed to many causes, including ageing populations, changing disease patterns in the population and evolving societal norms and values in society, some of which have yielded higher expectations for access to primary care, improved patient experience and increased patient involvement in care decision making [19, 39–42]. The workload has also been affected by a shift in tasks from secondary to primary care, which has not always been accompanied by sufficient resources. Primary care increasingly manages conditions previously handled by secondary care, e.g. palliative care and chronic disease, and patients are discharged to primary care more quickly than before [43].

Many of the physicians’ statements concerned restricted autonomy and the burden of administrative work. Again, these findings are consistent with many international studies concerning physicians’ working conditions [6, 44–46]. Issues related to limited autonomy and administrative work burden for health care professionals have often been attributed to New Public Management (NPM) principles because physicians and other health care professionals are expected to document their work, take on administrative tasks and participate in management-led quality improvement initiatives to achieve organizational goals [24, 47, 48]. There has been a lively public debate in Sweden on NPM, with many physicians critiquing core NPM principles and highlighting the consequences for health care professionals [49–51]. In response to the criticism of NPM principles, the Swedish government has recently introduced the concept of “trust-based governance”, intended to reduce the administrative burden and “letting professionals be professional” by allowing them to focus on their core activities, primarily patient work [52, 53]. This initiative is new and there are no research studies on the concept yet to examine whether or how it can be realized in practice.

Three types of rewards emerged from the analysis. The physicians found the content of their work to be stimulating, their job to be meaningful because their work is important for patients and the work climate to be supportive. These findings are aligned with other studies of the physician profession in other countries, many of which have shown the relevance of physicians’ personal sense of competence [54], collegial relationships [55] and patient interaction [56] for their job satisfaction.

Two of the three types of rewards in our study, stimulating work content and meaningfulness of the work, are consistent with so-called motivating factors in Herzberg’s Two Factor Theory. The theory posits that the presence of motivators such as achievement, recognition, responsibility and advancement create satisfaction by fulfilling individuals’ needs for meaning and personal growth [57]. The third type of reward in our study, a supportive work climate, is considered a hygiene factor in Herzberg’s theory. The presence of such factors does not necessarily build motivation. Rather, hygiene factors operate primarily to dissatisfy employees when they are not present [58].

Overall, we found the ERI model to be useful to explore physicians’ primary care work and working conditions and to identify efforts and rewards as well as approaches to managing effort/reward imbalances of relevance for their job satisfaction. Most of the efforts and rewards that emerged from our analysis are in line with issues addressed in questionnaires based on ERI. However, our study yielded few statements by the physicians about issues such as their income, employment security, job promotion prospects or whether the position adequately reflected their education and training, all of which are issues included in ERI-based questionnaires [34]. The paucity of these issues in our interviews suggests that the applicability of the ERI model depends on the type of work or professions being studied.

Few physicians mentioned income as a reward that contributed to their job satisfaction. We did not specifically ask the physicians if they considered their income to be a reward or an incentive that could offset the efforts they perceived. Speaking openly about one’s wage is usually considered inappropriate in Sweden. This reluctance has been attributed to the so-called Jantelagen, i.e. a widely held attitude of disapproval towards expressions of individuality or personal success [59]. Further, while physicians’ income may be a motivating factor for choosing the occupation, the salary is unlikely to be decisive for job satisfaction. Salary is a hygiene factor in Herzberg’s Two Factor Theory, meaning that it can lead to dissatisfaction but when fully catered for, is not sufficient to satisfy employees [58].

Coping with effort/reward imbalance in the ERI model is described as over-commitment, which means sacrificing a great deal for one’s work and/or seeking approval [34]. Items in the ERI-based questionnaire on over-commitment concern being overwhelmed by work, having difficulties switching off work and sacrificing too much for the job. Such consequences were mentioned by the physicians in our study, but not as approaches to handle effort/reward imbalances. Instead, the physicians viewed job enrichment, e.g. working with digital patient consultations, as an approach to manage such imbalances. Another coping approach was to use decisional latitude to achieve a more reasonable workload. Both approaches reduced the physicians’ total workload, enhanced their autonomy and improved their work/life balance. The results suggest that the responsibilities are placed on the physicians themselves to deal with imbalances in their work situation.

There are a number of limitations which should be considered in interpreting the findings of this study. The transferability of our results is limited to primary care settings in Sweden and the study findings cannot be directly transferred to international settings. Regardless, the study results may be relevant for other settings because the sample was adequate [60]. We sought analytical generalization rather than statistical generalization by comparing findings with comparable empirical research and relevant theories.

There are also considerable strengths to the study. The credibility of the study was enhanced by the multidisciplinary research team (see Methods) as this composition of researchers facilitated different perspectives on the investigated issue [32]. The number of interviews (*n* = 21) was relatively high, which was another strength of the study. Research suggests that the more information power, i.e. information relevant for a study, a sample holds, the fewer interviews are needed. Information power depends on the study aim, sample specificity, use of an established theory or framework, quality of the dialogue of the interviews and the analysis strategy [61]. According to Guest [62], 12 interviewees should be sufficient if the informants are knowledgeable about the subject, data quality is satisfactory and the aim is to understand common perceptions and experiences rather than to assess variation between groups. All the participants in our study had experience of working as physicians in primary care, the interviews followed the outline of the interview guide and the interviews provided information-rich material. We used quotations from 15 different participants, something which added to the transparency and trustworthiness of the findings. The fact that the participants came from different geographic regions of Sweden and from both public and private organizations was another strength. Furthermore, both men and women of different ages were included and they differed with regard to previous experiences from primary care work.

## Conclusions

In conclusion, primary care physicians perceive numerous types of efforts in their job, which is consistent with research concerning work stress and associated consequences, such as poor subjective health and well-being. There are also rewards according to primary care physicians, but the findings suggest a lack of reciprocity in terms of efforts and rewards although firm conclusions cannot be drawn since the study did not investigate the magnitude of the various efforts and rewards or the effectiveness of the approaches the physicians use to cope with imbalances. We found the ERI model to be useful to explore physicians’ primary care work and working conditions but its applicability likely depends on the type of work or professions being studied.

## Data Availability

The data will be available from the corresponding author on reasonable request. Transcripts are stored in the authors’ password-protected computers and no unauthorized persons have access to the data.
